# Comparison between dynamic contour tonometry and Goldmann applanation tonometry correcting equations

**DOI:** 10.1038/s41598-022-24318-y

**Published:** 2022-11-23

**Authors:** Maddalena De Bernardo, Claudia Casaburi, Ilaria De Pascale, Luigi Capasso, Ferdinando Cione, Nicola Rosa

**Affiliations:** 1 Department of Medicine and Surgery, University of Salerno, Via Salvador Allende, 84081 Baronissi, SA Italy; 2 A.S.L. NA1 Centro, Naples, Italy

**Keywords:** Ocular hypertension, Eye diseases

## Abstract

In order to investigate the reliability of correcting GAT formulas in comparison with dynamic contour tonometry (DCT), this study included 112 right eyes of 112 healthy subjects aged from 21 to 77 years, whose eyes underwent to a full ophthalmologic exam. IOP was measured in each eye with DCT and then with GAT. IOP values obtained with GAT were corrected with 10 equations and then compared with those provided by DCT. Participants mean age was 42.24 ± 14.08 years; mean IOP measured with DCT was 17.61 ± 2.87 and 15.50 ± 2.47 mmHg, measured with GAT. The mean discordance between DCT and GAT measurements was 2.11 ± 2.24 mmHg. All the correcting formulas, but Srodka one (*p* ˂ 0.001), tend to increase the difference between GAT and DCT. According to these results Śródka equation provides the best correction, reducing the difference between the two IOP measurement methods of − 0.03 ± 0.85 mmHg. Other equations do not provide a valid improvement of the agreement between the two methods or they provide a worsening of the agreement.

## Introduction

The intraocular pressure (IOP) measurement is crucial not only in the diagnosis and in management of glaucoma, as IOP represents the only treatable risk factor in clinical practice, but it is also essential in the post-operative management of corneal, cataract and vitreo-retinal surgeries and to help the detection of some rare diseases^1^.

Goldmann applanation tonometry (GAT), based on the so called Imbert-Fick law^2,3^, is the gold standard for IOP measurement, but unfortunately several factors, such as central corneal thickness (CCT), curvature (Km) and structure can influence its accuracy^4^.

It is well known that in case of cataract surgery after corneal refractive procedures, the intraocular lens (IOL) power to be implanted is underestimated^5–8^, as well as the GAT measurements are absolutely unreliable^9^.

To obtain more reliable GAT values, several experimental and theoretical calculation-based studies elaborated correcting formulas, based upon different parameters influencing GAT readings, such as corneal CCT, Km and subject age^10–18^.

The problem of testing the accuracy of these formulas is that results have to be compared with the true IOP value, measurable in vivo with an invasive technique, which is inexecutable in ordinary clinical practice.

Quite a few years ago, a new tonometer has been launched into the market, namely the dynamic contour tonometry (DCT); it is based on the Pascal law^19^, as an alternative of applanation principle, and it seems to be not influenced by CCT, Km, stiffness, properties and morphology^20–22^.

As DCT is assumed to be independent from corneal characteristics, the aim of this study is to compare DCT measurements to GAT, corrected according to 10 different formulas (which take into account some corneal parameters) in healthy subjects, to verify if these formulas effectively provide GAT values not influenced by corneal properties.

## Methods

One hundred and twelve right eyes of 112 healthy patients with no history of corneal diseases, previous corneal/ocular surgery or trauma, were included in this retrospective study, performed in 2021 on data previously obtained. Patients’ age ranged from 21 to 77 (mean = 42.24 ± 14.08) years old. The present study adhers to the ethical principles of the Declaration of Helsinki. Informed consent was obtained from each patient, and Institutional Review Board (IRB) approval was also obtained from the ComEtico Campania Sud (CECS). All the patients underwent a complete ophthalmic examination including, among the others, a CCT and Km measurement with a Pentacam (Oculus, Germany); the examination was considered to be reliable when the quality factor was 95% or higher, as suggested by the factory. Corneas were anesthetized with oxybuprocaine eye drops [Novesina, Novartis, Italy] before IOP measurements. During the measurement, subjects were asked to keep both eyes opened, breathe quietly, and fixate into the distance behind the examiner.

IOP measurement were taken in the same order on the same day, between 3:00 and 5:00 p.m. DCT was performed first, followed 10–15 min later by GAT and each of them was performed once, to avoid IOP reduction due to repeated measurement^4^. DCT head consists in a cylindrical tip with a surface that resembles the corneal contour when the pressure on both corneal sides is equal, so there is no need to apply additional forces that could modify the corneal profile and to influence the following GAT IOP measurements^19^.

When the tip of the DCT tonometer contacts the cornea, an audible signal, that changes in pitch with variations in pressure detected, indicates the correct positioning. The tip generates an electrical signal, proportional to the IOP value, which is calculated automatically and displayed on the digital screen along with the ocular pulse amplitude and the quality of each reading with a scale classified from Q1 (optimum) to Q5 (unacceptable). Only good quality DCT readings (Q1, Q2, and Q3) were used for this study. Regarding GAT measurements, a fluorescein strip was used in each eye and the patient was asked to blink several times before the measurement was performed, to obtain a fluorescein ring thickness similar to the one shown in the Goldmann tonometer manual, that should be between 0.25 and 0.3 mm. Patient comfort was ensured and the examiner did not hold the lids open, to avoid any pressure on the globe. DCT and GAT measurements were obtained by 2 different examiners who were not aware of results obtained with the other technique.

IOP measurements provided by GAT have been corrected by using the 10 formulas shown in Table 1, derived from manometric studies in vivo^16,17^, from retrospective studies^11^ and methanalisys^15^, and from studies based on mathematical calculation^10,12–14^.

**Table 1:** informations about GAT correcting formulas used.

Author	Year	n° eyes	Formulas: IOP_corrected =_	Type of study
Shimmyo^8^	2003	1482	(1) IOP_Gat_ + (550 − CCT)/18^–0.005^*^IOPgat^	Retrospective study (Refractive surgery eyes)
ShimmyoR^9^	2003	1482	(2) IOP_gat_ + (550 − CCT)/18^–0.005^*^IOPgat^ + 0.8(R − 7.848837)	Retrospective study (Refractive surgery eyes)
Elsheikh 2009^8^	2009	–	(3) IOP_Gat_/(A_CCT*_A_R*_A_age*_A_Iop)_	Numerical study on corneal model
Elsheikh 2011^10^	2011	–	(4) IOP_Gat_/(A_CCT1*_A_R1*_A_age1*_A_Iop1)_	Numerical study on corneal model
Śródka^11^	2013	–	(5) [*e*(CCT_c_ − (R/R_c_CCT) + 1] (IOP_CA_ + 3) − 3 CCT_c_ = 0.550 mm R_c_ = 7.8 mm *e*: 1/mm IOPca = − 1.61 + 0.94 IOPgat + 0.011 IOPgat	Study based on calibration function and on R and CCT effect experimental determined
Chihara^12^	2007	–	(6) $\frac{{{\text{IOP}}_{{{\text{Gat}}}} + {4}.{15}}}{{{19}.0{9}*{\text{CCT}}^{{2}} /{\text{A}}\left( {{\text{R1}}0^{{3}} - {\text{CCT}}/{2}} \right){1}0^{{4}} }} + 1$ where A is assumed to be constant = 0.34mm^2^	Based on theoretical model
Doughty^13^	2000	600	(7) IOP_Gat_ + 25[(545 − CCT)545]	Methanalysis (chronical deases eye)
Foster^14^	2000	23	(8) (IOP_Gat_*1.08) + 5.5	Manometric study in vivo (chinese population)
Kohlhaas^15^	2006	125	(9) IOP_Gat_ + ΔIOP where ΔIOP = (− 0.0423*CCT) + 23.28	Manometric study in vivo. The equation is based on Dresdner table that establishes an increase of ± 1 mmHg for each 25 µm of variation from the “normal” CCT value (550 µm)
Ehlers^16^	1975	29	(10) IOP_Gat_ + CF CF = 0.071*[520 − CCT*(IOP_Gat_ − 20)]*(IOP_Gat-20_) + 1]	Manometric study in vivo that establishes an increase of ± 0.71 mmHg for each 10 µm of variation from the “normal” CCT value of 550 µm

Statistical analysis was performed with SPSS 26.0 (SPSS, Inc., Chicago, IL). The normality of data was examined by the exact Kolmogorov–Smirnov. The Student T-test was used for pair-wise comparisons of IOP obtained with DCT and GAT and also with DCT and all IOP obtained after applying 10 correcting formulas. This latter test was also used for pair-wise comparison of differences between measurement obtained with and without IOP correction.

A p-value less than 0.05 was considered statistically significant. Therefore, Bland–Altman evaluation, was performed.

Required sample size was calculated with a power calculation software (G*Power, Version 3.1.9.6, Faul, Erdfelder, Lang, & Buchner, 2020. Available at https://www.gpower.hhu.de). It was estimated that with a significance level of 5% and a test power of 80%, a sample size of 73 eyes would be necessary to detect a mean IOP difference of 0.05 mmHg, given a within-subject SD for IOP equal to 1.50 mmHg.

The differences were evaluated both as absolute and non-absolute error utilizing SPSS.

## Results

All analyzed data were normally distributed (all *p* > 0.050). The obtained results are summarized in Tables 2, 3, 4. The correlation between uncorrected and corrected GAT with DCT is shown in Figs. 1, 2, 3, 4, 5, 6, 7, 8, 9, 10 and 11.

**Table 2:** DCT and GAT (corrected with different formulas) difference in mmHg.

	Mean ± SD	*p*
DCT-GAT_uncorrected_	2.11 ± 2.24	*p* < 0.001
(5)DCT-GAT_Śródka_	1.86 ± 2.48	*p* < 0.001
(1)DCT-GAT_Shimmyo_	1.97 ± 2.62	*p* < 0.001
(9)DCT-GAT_Kohlhaas_	1.98 ± 2.46	*p* < 0.001
(2)DCT-GAT_ShimmyoR_	2.02 ± 2.80	*p* < 0.001
(7)DCT-GAT_Doughty_	2.22 ± 2.52	*p* < 0.001
(3)DCT-GAT_Elsheik2011_	3.13 ± 2.36	*p* < 0.001
(10)DCT-GAT_Ehlers_	4.13 ± 2.95	*p* < 0.001
(4)DCT-GAT_Elsheik2009_	4.27 ± 2.65	*p* < 0.001
(6)DCT-GAT_Chihara_	− 2.04 ± 2.24	*p* < 0.001
(8)DCT-GAT_Foster_	− 4.63 ± 2.30	*p* < 0.001

*DCT* Dynamic contour tonometry, *GAT* Goldmann applanation tonometry, *SD* Standard deviation, *p* Level of significance according to paired T-test.

**Table 3:** DCT and GAT (corrected with different formulas) differences expressed in absolute value in mmHg.

	Mean |DCT-GAT| in mmHg ± SD	*P* value
DCT-GAT_uncorrected_	2.43 ± 1.89	*p* < 0.001
(5)DCT-GAT _Śródka_	2.40 ± 1.95	*p* < 0.001
(9)DCT-GAT_Kohlhaas_	2.51 ± 1.92	*p* < 0.001
(6)DCT-GAT_Chihara_	2.56 ± 1.62	*p* < 0.001
(1)DCT-GAT_Shimmyo_	2.61 ± 1.97	*p* < 0.001
(7)DCT-GAT_Doughty_	2.68 ± 2.02	*p* < 0.001
(2)DCT-GAT_ShimmyoR_	2.76 ± 2.07	*p* < 0.001
(3)DCT-GAT_Elsheik2011_	3.29 ± 2.13	*p* < 0.001
(10)DCT-GAT_Ehlers_	4.29 ± 2.70	*p* < 0.001
(4)DCT-GAT_Elsheik2009_	4.32 ± 2.58	*p* < 0.001
(8)DCT-GAT_Foster_	4.69 ± 2.16	*p* < 0.001

*DCT* Dynamic contour tonometry, *GAT* Goldmann applanation tonometry, *SD* Standard deviation, *p* Level of significance according to paired T-test.

**Table 4:** Results provided by different authors and by the current study after the application of equations correction.

Author	n° eyes	Glaucoma	Chihara (6)	Ehlers (10)	Elsheikh 2009 (3)	Elsheikh 2011 (4)	Kohlhaas (9)	Srodka (5)	Doughty and Zaman (7)	Foster (8)	Shimmyo (1)	ShimmyoR (2)
Asejczyk-Widlika et al.^25^	108	A 19 < P < 20	↑	↑	↓		↑	↓			↑	
		B 21 < P < 29	↑	↓	↓		↑	↓			↓	
		C 30 < P < 42	↑	↑	↓		↑	↓			↑	↑
Ghee et al.^24^	135 65	Glaucoma suspect		↑			↑		↑	↑	↑	
		Glaucoma group		↓			↓		↓	↑	↓	
Wachtl et al.^26^	105	Glaucoma subjects		↑	↑	↑	↓					
Current study	112	No	↑	↑	↑	↑	↑	↓	↑	↑	↑	↑

↑ = difference increase between the two methods (GAT and DCT) after correcction: poor usefull equation, ↓ = difference decrease between two methods (GAT and DCT) after correction: usefull equation. P = intraocular pressure, provided by GAT and expressed in mmHg.

In Asejczyk-Widlika et al.^25^ study subjects were divided in 3 groups (A, B, C) according to IOP values. In Ghee S et al.^24^ study subjects were divided in two groups (glaucoma suspect and glaucoma group).

**Figure 1:**
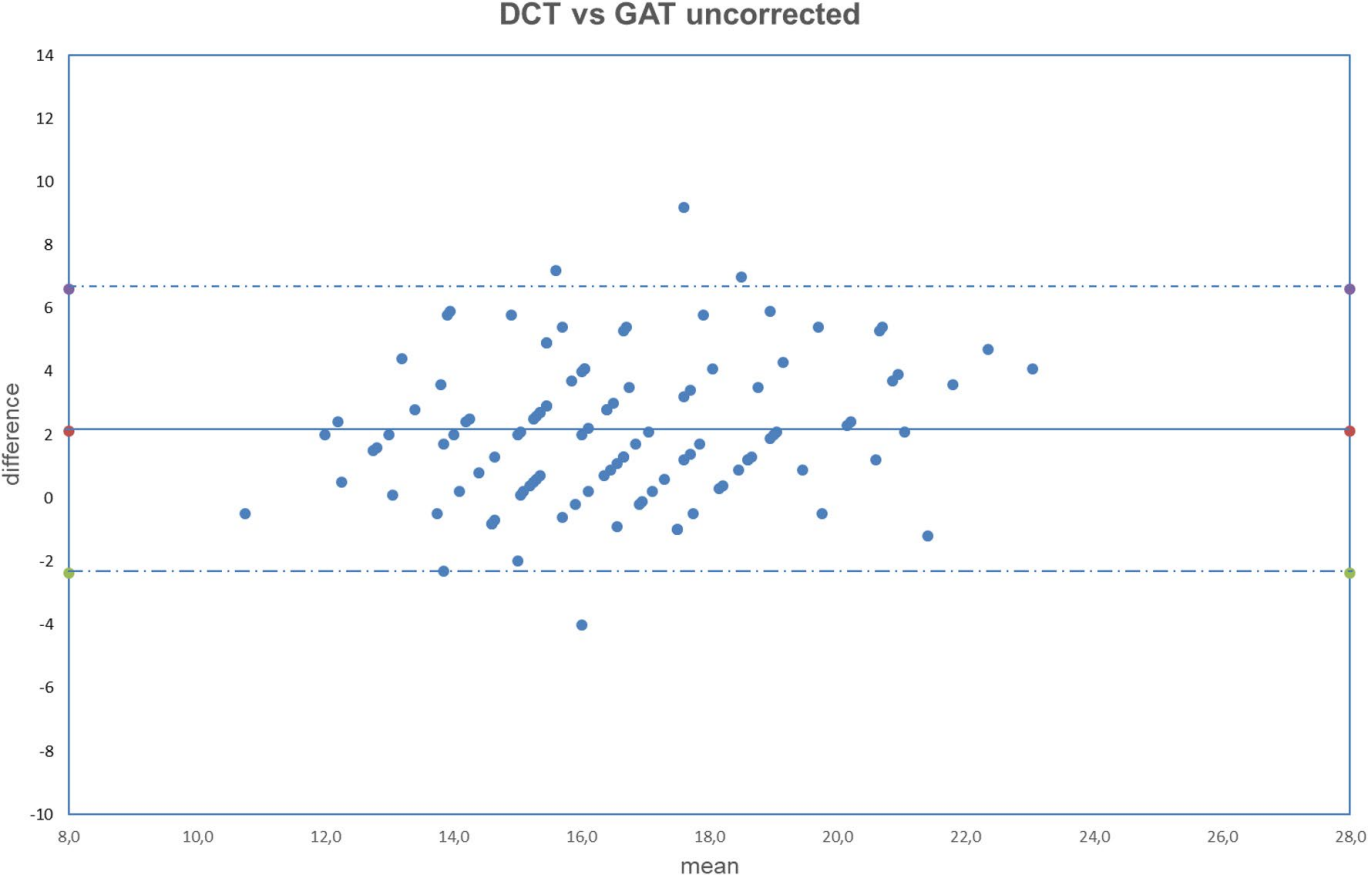
Bland–Altman plot between IOP measured with DCT and GAT uncorrected. Dashed line: mean difference. Dash-dotted lines: mean difference (2.11 mmHg) ± 2 standard deviation (2.24 mmHg) of the differences.

**Figure 2:**
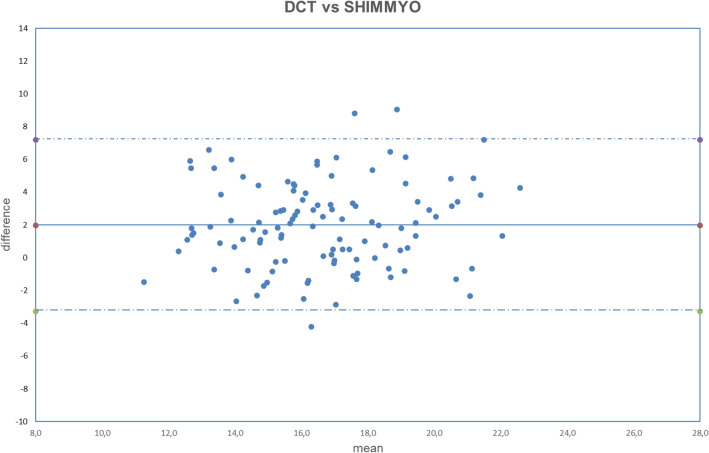
Bland–Altman plot between IOP measured with DCT and GAT corrected with Shimmyo formula. Dashed line: mean difference. Dash-dotted lines: mean difference (1.97 mmHg) ± 2 standard deviation (2.62 mmHg) of the differences.

**Figure 3:**
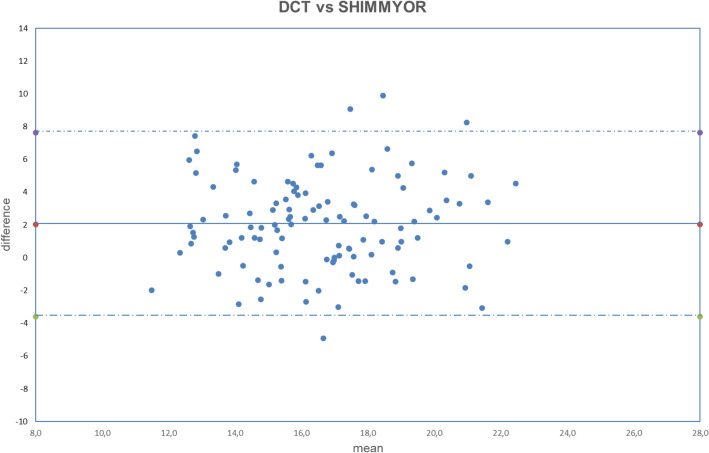
Bland–Altman plot between IOP measured with DCT and GAT corrected with ShimmyoR formula. Dashed line: mean difference. Dash-dotted lines: mean difference (2.02 mmHg) ± 2 standard deviation (2.80 mmHg) of the differences.

**Figure 4:**
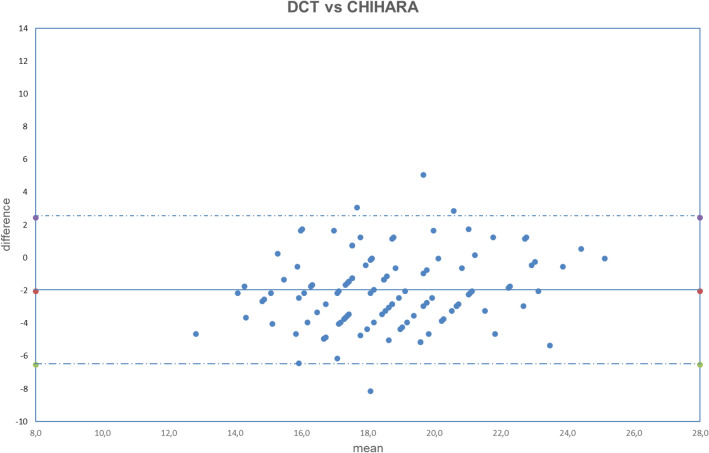
Bland–Altman plot between IOP measured with DCT and GAT corrected with Chihara formula. Dashed line: mean difference. Dash-dotted lines: mean difference (− 2.04 mmHg) ± 2 standard deviation (2.24 mmHg) of the differences.

**Figure 5:**
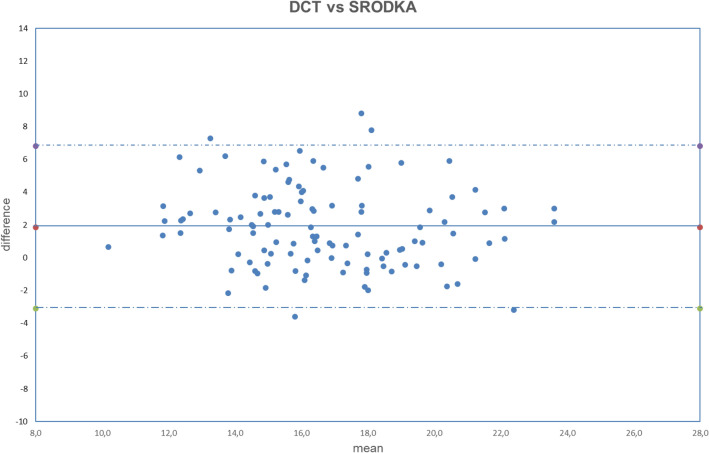
Bland–Altman plot between IOP measured with DCT and GAT corrected with Śródka formula. Dashed line: mean difference. Dash-dotted lines: mean difference (1.86 mmHg) ± 2 standard deviation (2.48 mmHg) of the differences.

**Figure 6:**
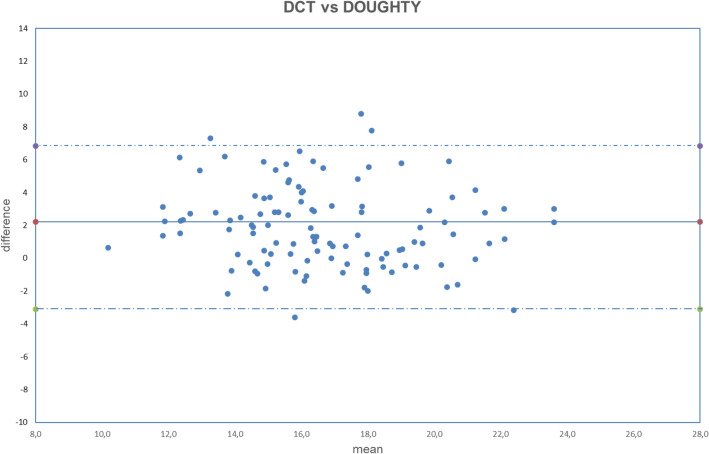
Bland–Altman plot between IOP measured with DCT and GAT corrected with Doughty formula. Dashed line: mean difference. Dash-dotted lines: mean difference (2.22 mmHg) ± 2 standard deviation (2.52 mmHg) of the differences.

**Figure 7:**
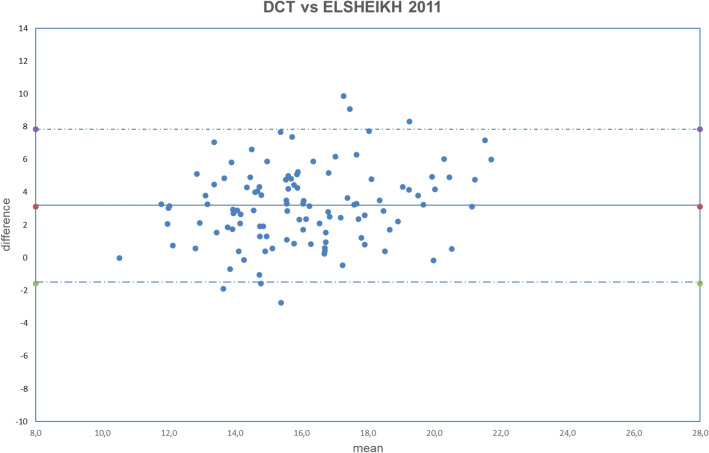
Bland–Altman plot between IOP measured with DCT and GAT corrected with Elsheikh2011 formula. Dashed line: mean difference. Dash-dotted lines: mean difference (3.13 mmHg) ± 2 standard deviation (2.36 mmHg) of the differences.

**Figure 8:**
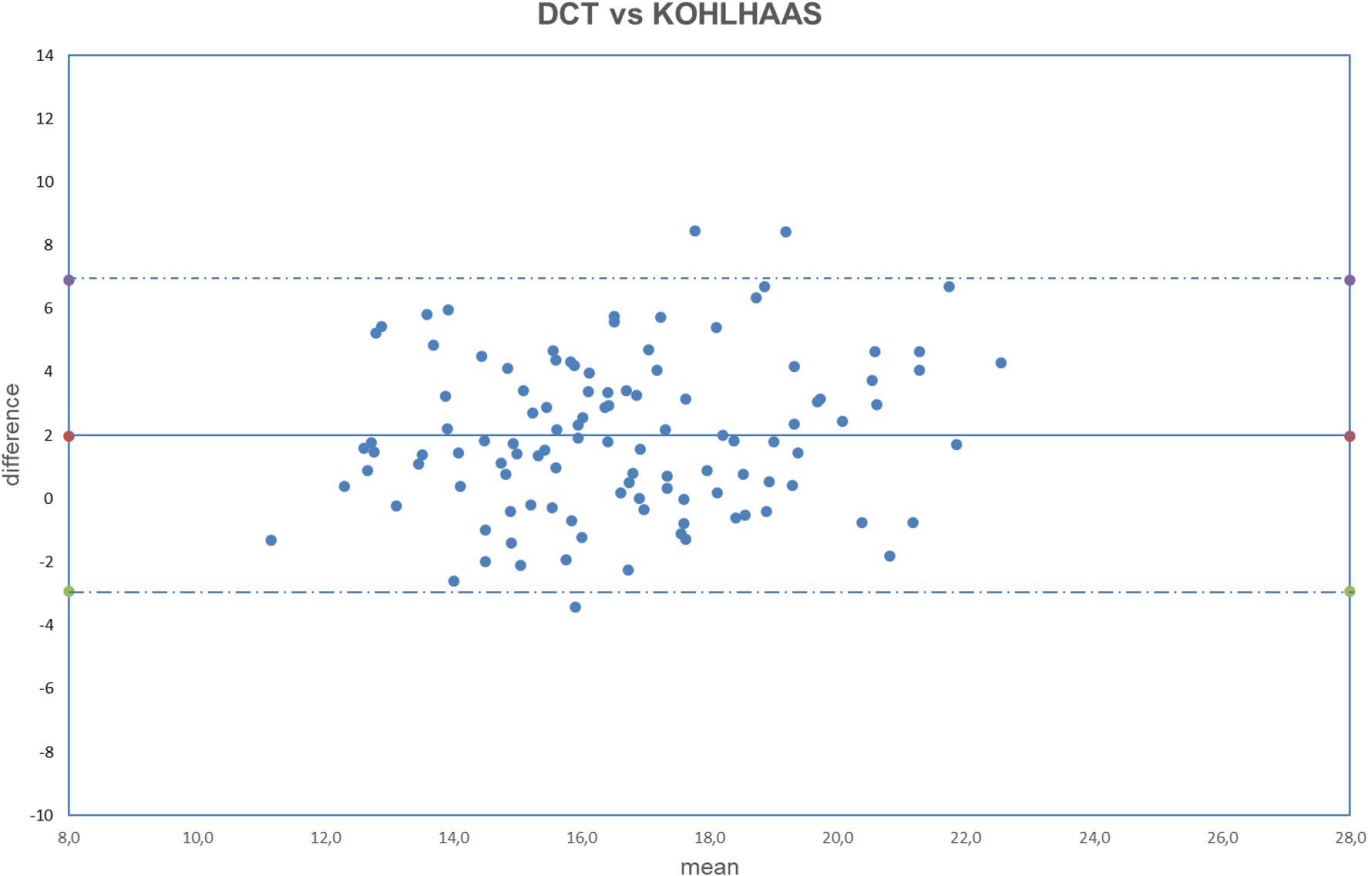
Bland–Altman plot between IOP measured with DCT and GAT corrected with Kohlhaas formula. Dashed line: mean difference. Dash-dotted lines: mean difference (1.98 mmHg) ± 2 standard deviation (2.46 mmHg) of the differences.

**Figure 9:**
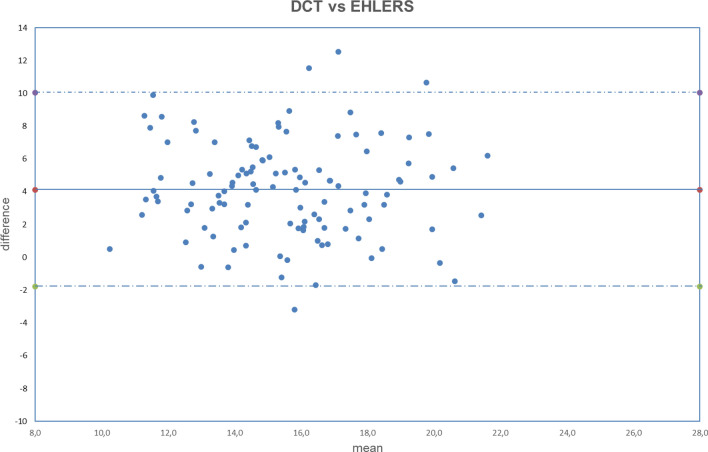
Bland–Altman plot between IOP measured with DCT and GAT corrected with Ehlers formula. Dashed line: mean difference. Dash-dotted lines: mean difference (4.13 mmHg) ± 2 standard deviation (2.95 mmHg) of the differences.

**Figure 10:**
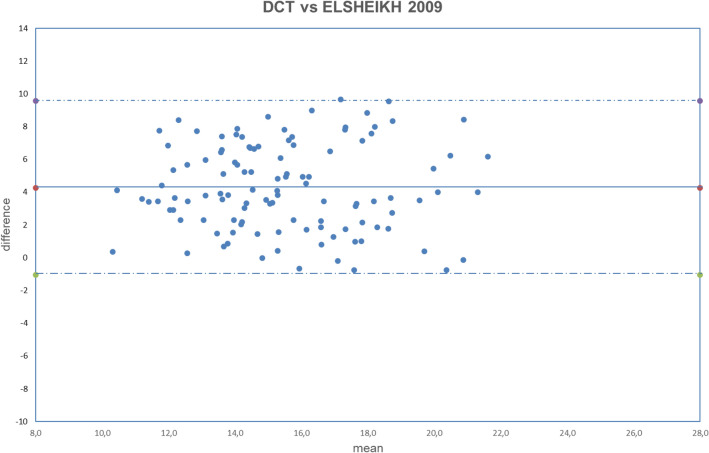
Bland–Altman plot between IOP measured with DCT and GAT corrected with Elsheikh2009 formula. Dashed line: mean difference. Dash-dotted lines: mean difference (4.27 mmHg) ± 2 standard deviation (2.65 mmHg) of the differences.

**Figure 11:**
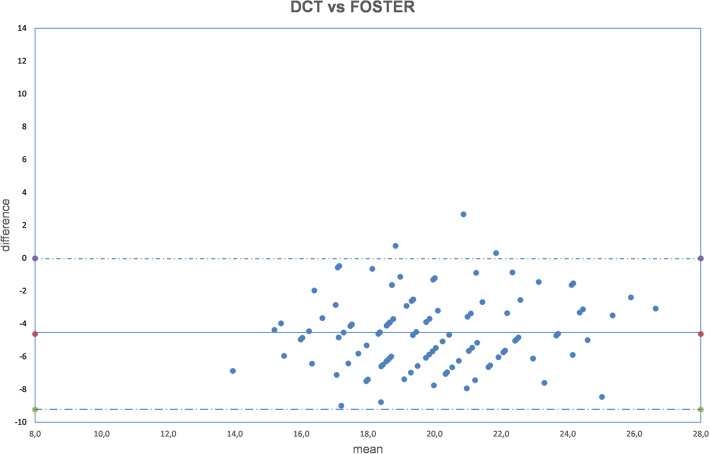
Bland–Altman plot between IOP measured with DCT and GAT corrected with Foster formula. Dashed line: mean difference. Dash-dotted lines: mean difference (− 4.63 mmHg) ± 2 standard deviation (2.30 mmHg) of the differences.

Both fixed bias and proportional bias were found only with Foster correction (Fig. 11). There was no evidence of fixed bias or proportional bias with all other correction formulas (Figs. 1, 2, 3, 4, 5, 6, 7, 8, 9 and 10).

According to these results, IOP measurements provided by GAT without any correction are lower than those provided by DCT.

Utilizing formulas shown in Table 1, an increase of mean difference (DCT-GAT_corrected_) is obtained with formulas number 3, 4, 7, 8 and 10; in particular, formulas 3, 4, 7, 10 tend to underestimate the IOP measured by GAT, providing a further reduction of IOP value in comparison to DCT. Formulas 6 and 8, instead, tend to overestimate the IOP measured by GAT: in these cases, the difference DCT-GAT_corrected_ is negative, since GAT corrected by equations 6 and 8 is higher than DCT.

As shown in Table 2, a reduction of mean difference between DCT and GAT_corrected_ is obtained with formulas 1, 2, 5, 7 and 9 formulas; Śródka (formula 5) provides the highest reduction of mean difference (DCT-GAT_Srodka_ = 1.86 ± 2.48).

If we consider the absolute error (Table 3) we can clearly see that all the formulas, but Śródka one, tend to increase the difference between GAT and DCT. Śródka equation provides a statistically significant decrease of the difference between these two methods.

## Discussion

IOP role in etiopathogenesis and development of glaucoma has been recently questioned, in fact it is not included in primary open-angle glaucoma definition given by European Glaucoma society (EGS) guidance. Nevertheless, several studies have established the importance of its reduction in glaucomatous patient management^23^. Therefore, tonometry remains mandatory in all the patient over 50 years old in all the subjects considered to be at risk of glaucoma development.

Even if GAT is considered to be the gold standard, but unfortunately it cannot be used to measure the IOP in different body’s position^24^ and several factors make it not reliable; in fact it is based on the so-called Imbert-Fick law that is not a physical law, or even an engineering principle, but it was just invoked as an explanation of how applanation tonometers worked^2,3^.

The validity of the law needs the structure applanated by the flat surface to be thin, elastic, flexible, and the pressure of the applanating surface to be the only force acting against it. Unfortunately, the cornea is aspherical, wet and neither perfectly flexible nor infinitely thin.

Moreover, tonometer tips do not contact the cornea alone, but come into contact with the precorneal tear film, which creates surface tension and produces capillary attraction (or repulsion) between it and objects in contact with its meniscus.

For this reason, it is clear that corneal thickness influences IOP measurement; with thin corneas causing wrong low readings and thick corneas wrong high measurement if the thickness is the result of increased collagen fibrils; low readings if the thickness is a result of corneal edema^4,25^.

Several authors tried to overcome these problems, proposing different correcting formulas.

We decided to compare the results obtained with these formulas with DCT because this tonometry instrument is based on Blaise Pascal’s law of hydrostatic pressure, in which a hypothetical corneal shape (contour) is achieved when the pressure on either side of the cornea is equal.

The force distribution that is needed to gently fit the corneal surface to that hypothetical contour counterbalances the force distribution generated by the IOP. A tonometer tip, equipped with this hypothetical contour and that touches the cornea, alters the corneal shape into the desired contour. The distribution of interface forces between the tip and the cornea equals the force distribution generated by the IOP. Hence, a pressure sensor that is centrally and concavely embedded into the tonometer tip should precisely measures the pressure of the eye.

Moreover, several authors found a good agreement between intra-camerural IOP and IOP provided by DCT, with a lower influence of central corneal thickness on DCT readings in comparison to GAT readings^20,22^.

DCT seems to provide IOP readings that should be closer to the “real” pressure, but it is not widely spread and it requires an higher patient compliance.

Several papers compared the results obtained with GAT and DCT^26–28^, but only few tried to correlate the IOP readings with correcting formulas.

Our results are in line with previous studies where has been established that DCT provides higher value of IOP in comparison to GAT^24,27,28^. In particular the mean difference between DCT and GAT (2.11 mmHg) and the range (from − 4 to 9 mmHg) are consistent with Cook et al.^26^, who found a mean difference of 1.8 mmHg and a range from − 2.9 to 6.5 mmHg.

Concerning the comparison between DCT and GAT correcting formulas^29–31^, our results aren’t always in agreement.

Results disagreement could be due to the different characteristics of the examined population, in particular it could be related to the different range of IOP, in fact in our study healthy subjects were considered, in the previous studies patient with glaucoma or with glaucoma suspect were examined.

For example, Asejczyk-Widlicka et al.^30^ found Śródka equation and Elsheikh2009 equation to be useful in IOP measurements, opposite to other formulas which did not provide a better agreement. In our study, just Śródka equation is associated with a statistically significant decrease in the differences between GAT and DCT, providing a better correction. These differences could be related to the different considered GAT IOP values interval, that in our study ranged between 11 and 22 mmHg, while in Asejczyk-Widlicka et al.^30^ study it ranged between 19 and 42 mmHg.

This study has some limitations: both DCT and GAT measurements were performed once, to avoid IOP reduction due to repeated measurement^4^, even if in comparative studies of IOP, it is common practice to take the average of three measurements. The equations used in this study come from different theoretical models experimental and manometric studies, but they do not provide an important improvement of the agreement between GAT and DCT in fact some of them provide a worsening of the agreement. Just Śródka equation slightely reduces the difference between DCT and GAT, but this is just a statistically significant result which doesn’t seem to justify its use and application in clinical practice.

Despite the results disagreement between the studies, the general tendency is a poor utility of the equations in all the studies.

This poor efficacy of equation could be due to the fact that the parameters considered by the equations could be not sufficient to explain the disagreement between the two methods of tonometry. Moreover, this study is based on the assumption that DCT provides values that are closer to the real IOP, assumption that is supported by some manometric studies. Nevertheless, it is also necessary to assume that DCT could not reflect the real intraocular pressure.

## Data Availability

The datasets generated and analyzed during the current study are available from the corresponding author on reasonable request.
